# Antispasmodic Effect of Essential Oils and Their Constituents: A Review

**DOI:** 10.3390/molecules24091675

**Published:** 2019-04-29

**Authors:** Simona Codruta Heghes, Oliviu Vostinaru, Lucia Maria Rus, Cristina Mogosan, Cristina Adela Iuga, Lorena Filip

**Affiliations:** 1 Department of Drug Analysis, Iuliu Hatieganu University of Medicine and Pharmacy, Cluj-Napoca 400349, Romania; cmaier@umfcluj.ro (S.C.H.); lrus@umfcluj.ro (L.M.R.); iugac@umfcluj.ro (C.A.I.); 2 Department of Pharmacology, Physiology and Physiopathology, Iuliu Hatieganu University of Medicine and Pharmacy, Cluj-Napoca 400349, Romania; cmogosan@umfcluj.ro; 3 Department of Proteomics and Metabolomics, MedFuture Research Center for Advanced Medicine, Iuliu Hatieganu University of Medicine and Pharmacy, Cluj-Napoca 400349, Romania; 4 Department of Bromatology, Hygiene, Nutrition, Iuliu Hatieganu University of Medicine and Pharmacy, Cluj-Napoca 400349, Romania; lfilip@umfcluj.ro

**Keywords:** essential oils, aromatic plants, antispasmodic effect, isolated ileum, monoterpenes

## Abstract

The antispasmodic effect of drugs is used for the symptomatic treatment of cramping and discomfort affecting smooth muscles from the gastrointestinal, billiary or genitourinary tract in a variety of clinical situations.The existing synthetic antispasmodic drugs may cause a series of unpleasant side effects, and therefore the discovery of new molecules of natural origin is an important goal for the pharmaceutical industry. This review describes a series of recent studies investigating the antispasmodic effect of essential oils from 39 plant species belonging to 12 families. The pharmacological models used in the studies together with the mechanistic discussions and the chemical composition of the essential oils are also detailed. The data clearly demonstrate the antispasmodic effect of the essential oils from the aromatic plant species studied. Further research is needed in order to ascertain the therapeutic importance of these findings.

## 1. Introduction

The antispasmodic (spasmolytic) effect of drugs is commonly used for the reduction of excessive smooth muscle contractility, responsible for cramping and discomfort in the abdominal area, caused by multiple conditions affecting the gastrointestinal, biliary or genitourinary tract [1]. Irritable bowel syndrome (IBS), biliary colic caused by gallstones, gastritis, colitis and pancreatitis or dysmenorrhea may affect large numbers of patients and usually require antispasmodic treatment for the relief of symptoms [1,2,3]. Additionally, antispasmodic compounds are also used for the reduction of discomfort caused by medical procedures like colonoscopy [4]. A variety of synthetic antispasmodic drugs have been authorized worldwide by the regulatory agencies, the most important being anticholinergic agents (butylscopolamine), direct smooth muscle relaxants (papaverine), calcium antagonists (pinaverium) or opioid receptor modulators (trimebutine) [1,2]. Despite their clinical efficacy, the use of these molecules is often limited by the development of unpleasant and sometimes severe side effects which may reduce patient compliance and impair treatment efficiency [1,4]. Historically, a long time before the golden age of medicinal chemistry, several aromatic plants were used in traditional medicine for the treatment of different ailments in some parts of the world. In Europe, aromatic plants like peppermint or thyme have been used for medical purposes since antiquity while in Chinese or Indian traditional medicine other aromatic species like cinnamon or sandalwood were known for centuries [5]. Nowadays, antispasmodic botanical remedies are used by a constantly increasing number of patients for symptomatic treatment of functional dyspepsia, intestinal, colonic or ureteral spasms, gallbladder hyperactivity and uterine cramps [6]. In the large category of medicinal plants, aromatic plants rich in essential oils are considered a valuable and easily accessible natural resource for the development of new molecules capable of becoming drug candidates. Essential oils are complex mixtures containing mainly aromatic terpenes classified in monoterpenes and sesquiterpenes according to the number of isoprene units but also phenylpropanoid compounds. These compounds are secondary metabolites formed by isoprenoid pathways in specialized secretory tissues of aromatic plants and diffused at the surface of their flowers or leaves [7]. The biological effects of essential oils have been extensively researched, as they can easily pass through cellular membranes and influence a variety of molecular targets from ion channels to intracellular enzymes [8]. Multiple in vitro and in vivo studies have confirmed the anti-oxidant, antimicrobial, antifungal, antiparasitary, anti-inflammatory, antinociceptive or antitumoral effects of essential oils [7], but the antispasmodic effect has been less studied experimentally, despite being mentioned in traditional medicine sources. Hence, this review was aimed at the investigation of the antispasmodic effect of essential oils, presenting the experimental models used for pharmacological testing with the subsequent mechanistic explanations, but also the chemical composition of the studied essential oils.

## 2. Methodology

A search was performed in Web of Science, PubMed and Scopus scientific databases, including the last 20 years (1998–2018). The search terms “essential oils” and “antispasmodic” (“spasmolytic”) were used for data selection. Only articles in English were included in this work. Our study investigated the antispasmodic effect of the essential oils and was not focused on the bronchodilator and vasodilator effects, presented by other reviews.

## 3. Results and Discussion

### 3.1. Preclinical Studies Investigating Antispasmodic Effect of Essential Oils

This review showed that essential oils from thirty-nine plant species belonging to twelve families presented antispasmodic properties demonstrated by specific animal models. The plants were organized alphabetically by family and botanical name, the proposed antispasmodic mechanism being also presented, where available. The families with the highest proportion of plant species showing antispasmodic effects due to essential oils (EO) were Lamiaceae (13 species), Apiaceae (6 species), and Asteraceae (5 species). Other identified families were Annonaceae and Poaceae (3 species), Rutaceae and Verbenaceae (2 species) while Anacardiaceae, Araceae, Geraniaceae, Rosaceae and Zingiberaceae families each presented only one plant species with antispasmodic essential oil (Table 1).

The majority of the presented preclinical studies used whole essential oils, the antispasmodic effect of individual chemical constituents of essential oils being rarely investigated. Sadraei et al. [28] demonstrated antispasmodic effects not only for the essential oil from *Melissa officinalis* but also for citral, one of its main components. Heimes et al. [31] investigated the spasmolytic effects of menthol, a major constituent in the essential oil from *Mentha x piperita*. De Souza et al. [33] tested the antispasmodic effect of several monoterpenes from *Mentha x villosa* essential oil, carvone and rotundifolone being the most active compounds. 

While several studies confirmed the antispasmodic effect of essential oils extracted from common vegetal species extensively used in Asia or Europe like *Cananga odorata* [10], *Foeniculum vulgare* [18] or *Artemisia dracunculus* [21], other studies showed significant spasmolytic effects of essential oils from less-known plant species like *Xylopia langsdorffiana* [12], *Ferula heuffelii* [17] or *Hofmeisteria schaffneri* [24], proving that new natural sources of bioactive molecules are constantly being discovered. 

The collected data from this review confirmed other studies investigating biological effects of essential oils. Martinez-Perez et al. [51] showed that monoterpenes frequently found in essential oils are the leading class of natural molecules responsible for the antispasmodic effect, followed by flavonoids and alkaloids. De Almeida et al. [52] and Sarmento-Neto et al. [53] also showed in their studies that aromatic plants from the Lamiaceae, Apiaceae and Asteraceae families are a rich source of essential oils, highly valuable for medicinal or industrial purposes. 

Also, analysis of the included studies showed that, generally, the experimental models used for the assesement of essential oil antispasmodic activity were represented by ex vivo techniques. Among these, the isolated guinea pig ileum method was preferred, being considered a precise pharmacological tool capable of investigating the antispasmodic effect of natural or synthetic compounds [54]. Other isolated organs used for the evaluation of antispasmodic effect were rabbit jejunum, rat ileum, bladder or uterus and sheep ruminal and abomasal muscles. An experimental model used in vitro cell cultures [15] and only two experiments used in vivo techniques: rabbit bladder in vivo and gastrointestinal transit test in mouse [10,24]. The ex vivo techniques are predominant due to their use without the limitations of drug bioavailability which may be a problematic issue for the in vivo models. Ex vivo methods are also well suited for mechanistic studies due to the diversity of contractile agents which could be used experimentally [55]. 

### 3.2. Clinical Studies Evaluating Antispasmodic Potential of Essential Oils

Antispasmodic effect of essential oils was investigated in several clinical studies for different situations: functional dyspepsia, irritable bowel syndrome, discomfort produced by endoscopic procedures, infantile colic or dysmenorrhea (Table 2). 

The analysis of data resulted from the presented clinical studies show that peppermint oil was the predominant essential oil used for symptomatic treatment of various conditions, the strongest evidence being available for irritable bowel syndrome (IBS). The randomized controlled studies of Cash et al. [60], Merat et al. [62] and Capello et al. [63] enrolled patients with IBS diagnosed according to Rome II or III criteria, showing that peppermint oil was superior to placebo in reducing the symptom score of irritable bowel syndrome (IBS) after oral administration for one or two months. According to other studies [58,59], peppermint and caraway essential oils are a possible treatment option for patients with functional dyspepsia, reducing epigastric discomfort and abdominal bloating over four weeks of treatment. Also, L-menthol and peppermint oil were tested for the reduction of discomfort caused by endoscopic procedures being used with good results in upper GI endoscopies, colonoscopies [66,67] but also in cholangiopancreatographies [68]. The study of Bezerra Alves et al. [69] found that *Mentha piperita* essential oil reduced the frequency of infantile colic. In addition, Ghodsi and Asltoghiri [70] found that fennel essential oil reduced primary dysmenorrhea symptoms after a prolonged oral administration [70]. 

The systematic reviews of Chumpitazi et al. [71] and Shams et al. [72] pointed out that peppermint oil was safe and well-tolerated by the patients, with a minimal side effect profile. Nevertheless, clinical studies evaluating the antispasmodic potential of essential oils have some limitations. They were represented mainly by randomized crossover or control studies using small numbers of patients with an insufficient statistical significance. Only the studies of Khanna et al. [61] and Pittler and Ernst [64] were metaanalysis with superior statistical power. Therefore, additional clinical studies are necessary to ascertain the therapeutic value of antispasmodic essential oils.

### 3.3. Mechanisms of Antispasmodic Effect of Essential Oils and Their Constituents

Smooth muscles are a key element present in the internal structure of multiple abdominal organs including stomach, intestine, bladder or uterus, receiving innervation from the autonomic nervous system but also autocrine or paracrine stimuli [73]. Recently, considerable progress was made to understand in great molecular detail the complex physiology of smooth muscle contraction. Excitation-contraction coupling occurs when Ca^2+^ ions enter from the extracellular side into the smooth muscle cells through sarcolemma voltage-dependent calcium channels, being also released from intracellular stores via inositol 1,4,5-triphosphate receptor (IP3R) situated on endoplasmic reticulum (ER) [74]. The calcium release from ER is triggered by the binding of agonists like acetylcholine or histamine on specific G-protein coupled receptors (GPCRs) from the membrane of smooth muscle cells, which activate phospholipase-C (PLC) to generate IP3. After the intracellular concentration of calcium has increased, Ca^2+^ ions bind to calmodulin (CaM) and phosphorylate the myosin light-chain kinase (MLCK) with the subsequent activation of the contractile apparatus [75]. 

**Figure 1:**
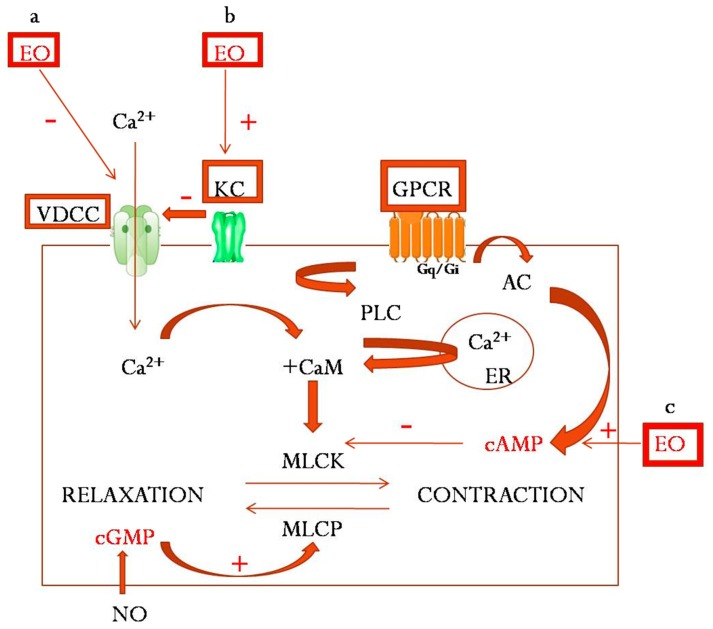
Main mechanisms of antispasmodic effect of essential oils: a. inhibition of voltage-dependent calcium channels; b. modulation of potassium channels; c. modulation of intracellular cAMP (EO—essential oil, VDCC—voltage-dependent calcium channel; KC—potasium channel, GPCR—G-protein coupled receptors, CaM—calmoduline, PLC—phospholipase C, AC—adenylyl cyclase, MLCK—myosin-light chain kinase, MLCP—myosin-light chain phosphatase, ER—endoplasmic reticulum, cGMP—cyclic guanosine monophosphate, cAMP—cyclic adenosine monophosphate, NO—nitric oxide).

Thus, the identification and characterization of multiple molecular targets involved in smooth muscle contraction has led to the development of a variety of drugs able to reduce excessive contractility responsible for cramps and colics of the abdominal organs. Among potential new drug candidates, essential oils have become increasingly attractive due to their complex chemical composition and multiple pharmacological mechanisms: inhibition of voltage-dependent calcium channels, modulation of potassium channels, antagonism of cholinergic receptors, and modulation of intracellular cyclic adenosine monophosphate (cAMP) (Figure 1). Although some details of the antispasmodic effect of essential oils and their constituents have been explained, further research is needed to better understand their mechanism of action at cellular and molecular levels.

#### 3.3.1. Inhibition of Voltage-Dependent Calcium Channels

The opening of voltage-dependent calcium channels (VDCCs) is directly responsible for Ca^2+^ influx into the smooth muscle cells, partially triggering the contractile mechanism. Thus, inhibition of VDCCs has a good potential of relaxing smooth muscles, already used by several antispasmodic drugs like pinaverium [76]. Essential oils have been studied for their antispasmodic effect, the inhibitory effect on voltage-dependent calcium channels being the most commonly reported mechanism of action in the studies mentioned in our review (19 studies from 39). Some of these studies presented detailed mechanistic explanations of the antispasmodic effect for the non-fractioned essential oils. Makrane et al. [37] showed that organic fractions rich in essential oils from *Origanum majorana* showed a consistent spasmolytic effect on isolated rat and rabbit ileum not altered by atropine, L-NAME or methylene blue, and shifted to the right the concentrations-response curves for CaCl_2_, suggesting a calcium channel blocking effect. Rasheed et al. [45] showed in an experiment on isolated rabbit jejunum that essential oil from *Rosa indica* relaxed the organ, shifting the calcium curves to the right, showing a similar effect to verapamil, a phenylalkylamine derivative from the calcium channel blocker class. The study of Souza et al. [35] identified the same mechanism for the essential oil from *Ocimum selloi* which reduced the contraction of isolated guinea pig ileum induced by carbachol, BaCl_2_ and K^+^ and shifted calcium concentration-response curves to the right. 

Other studies were focused on the individual components of the essential oils. Amato et al. showed that menthol (0.1 mM–30 mM) reduced in a concentration-dependent manner the contractility of human colon circular muscle, acting by an antagonistic effect on L-type Ca^2+^ channels [77]. The study of Ramos-Filho et al. [78] showed that menthol markedly inhibited contractions of wild and TRPM8 knockout mice bladder strips evoked by carbachol, CaCl_2_ or electric field stimulation. The effects of menthol were not influenced by previous incubation with sodium or potassium channel inhibitors or by the removal of urothelium, suggesting a blockade of calcium channels. Electrophysiological studies have shown that menthol was able to inhibit the calcium influx through the low-voltage activated Ca^2+^ channels but also to enhance the inactivation of high-voltage activated Ca^2+^ channels [79]. Another monoterpene, (-)-carvone was tested by Souza et al. [32] on guinea pig ileum in order to ascertain the mechanism of its spasmolytic effect, proving to be almost 100 times more potent than verapamil, a well-known calcium channel blocker (CCB) with a similar mode of action.Devi et al. [42] studied the antispasmodic effect of another terpene, citral, the major component of *Cymbopogon citratus* essential oil on rabbit ileum, the results showing a marked reduction of contractions evoked by CaCl_2_, similarly to verapamil. 

#### 3.3.2. Modulation of Potassium Channels

Potassium channels are largely distributed in human and animal tissues, having important physiological roles such as the regulation of smooth muscle tone [80]. Generally, the activation of voltage-gated potassium channels induces a hyperpolarisation of cell membrane with a subsequent de-activation of calcium channels leading to smooth muscle relaxation. Only a few studies investigated the effect of essential oils on potassium channels situated on smooth muscles of internal organs. Mehmood et al. [25] showed that a crude extract from *Matricaria chamomilla L.* containing sesquiterpenes (bisabolol) and flavonoids produced a significant antispasmodic effect on isolated rabbit jejunum. The effect on low K^+^ induced contractions was completely blocked by 4-aminopyridine, suggesting that the activation of potassium channels was responsible for smooth muscle relaxation. Khan et al. [40] showed that a crude extract from *Salvia officinalis* rich in essential oils (thujone, 1,8-cineole, camphor, linalool) caused a dose-dependent relaxation of isolated rabbit jejunum by an activation of K^+^ channels.

Silva et al. [81] investigated the mechanism of smooth muscle relaxation of rotundifolone, the major constituent of *Mentha x villosa* essential oil. Patch-clamp recordings made in mesenteric smooth muscle cells showed that rotundifolone significantly increased K^+^ currents, effect blocked by charybdotoxin which suggested the participation of big potassium (BK) channels. 

#### 3.3.3. Antagonism of Cholinergic Receptors

The parasympathetic nervous system has an important role in the regulation of gastrointestinal motility. Muscarinic receptors located directly on smooth muscle cells can trigger their contraction in response to acetylcholine, but nicotinic receptors have also been identified on the nerve cells from the enteric nervous system. Research on the effect of essential oils on cholinergic receptors from smooth muscles is extremely rare, only a few studies being published to date. Amato et al. [82] found that menthol induced relaxation of isolated mouse stomach by inhibiting nicotinic receptors from the enteric nervous system, reducing the release of acetylcholine from enteric nerves. The study of Lozon et al. [83] investigated the effects of vanilin, pulegone, eugenole, carvone, carvacrol, carveol, thymol, thymoquinone, menthone, and limonene on human nicotinic cholinergic receptors expressed in *Xenopus* oocytes. Carveol showed the most potent inhibition at the α 7 subunit of the nicotinic receptor. The molecular interactions between terpenic compounds from essential oils and nicotinic cholinergic receptors were investigated by electrophysiological studies which showed that menthol caused a shortening of channel open time and a prolongation of channel closed time of human α4β2 nicotinic receptors [83].

#### 3.3.4. Modulation of Intracellular Cyclic Adenosine Monophosphate (cAMP)

The main intracellular second messengers cAMP and cGMP are directly involved in smooth muscle relaxation. cAMP is generated by adenylyl cyclase mainly as a result of beta-adrenergic receptor activation. cGMP is produced by soluble guanylyl cyclase activated by nitric oxide or other mediators. Both cAMP and cGMP activate protein kinases PKA and PKG which may relax smooth muscles either by increasing the expulsion of calcium from the cell or by activation of MLC phosphatase which inhibits MLCK. The levels of cAMP and cGMP are severely reduced by the intervention of phosphodiesterases (PDE) involved in their degradation to inactive metabolites [73]. Multiple studies have investigated the effects of essential oils on the intracellular mechanisms of smooth muscle relaxation. In a study from 2018, Sandor et al. [22] studied the effects of the essential oil and extract from *Chamaemelum nobile L.* (roman chamomile) on isolated guinea pig ileum and rat gastrointestinal preparations. The essential oil significantly relaxed the isolated organs contracted with histamine, without any influence of a pretreatment with atropine, tetrodotoxin, propranolol or N^G^–nitro-L-arginine, thus suggesting an intracellular mechanism of the antispasmodic effect. Zavala-Mendoza et al. [23] investigated the antispasmodic mechanism of the essential oil from *Chrysactinia mexicana* on isolated rabbit ileum. The effect was reduced by the preincubation with dibutyryl-cAMP but increased by forskolin, whereas chelerytrine or L-NNA did not modify the response, suggesting an involvement of cAMP in the antispasmodic mechanism of the essential oil. The study of Kim et al. [10] showed that essential oil from *Cananga odorata* (ylang-ylang) relaxed the isolated rat bladder muscle, the effect being reduced by N-ethylmaleimide but not by inhibitors of NO pathway, demonstrating the involvement of cAMP. Also, Lis-Balchin and Hart [27] found that essential oil from *Lavandula angustifolia* induced relaxation of isolated guinea-pig ileum through a rise in intracellular level of cAMP The effects of individual components from essential oils on intracelullar mechanism of antispasmodic effect were less studied. Leal-Cardoso et al. [84] found that eugenol (1–2000 microM) relaxed the isolated rat ileum precontracted with KCl, without any influence from tetrodotoxin, L-NAME, hexamethonium or indomethacin, thus suggesting an intracellular mechanism.

### 3.4. Chemical Composition of the Essential Oils with Antispasmodic Activity

Essential oils are complex mixtures of volatile compounds with terpenoid or non-terpenoid structure that can be extracted from different parts of plants (flower, buds, seed, leaves, and fruits). Many of those compounds have been identified in essential oils and clasiffied as functionalized derivatives of alcohols, ketones or aldehydes, esters, ethers, oxydes and phenols [85,86]. The composition of an essential oil may vary according to the plant’s environment and growing conditions, stage of development, methods of harvesting, extraction, and storage. The major constituents of an essential oil can also vary according to different chemotypes of the same plant species [85]. Although there is a tendency to correlate pharmacological properties with the presence of certain functional groups, this concept cannot be generalized. Thus, neurotoxicity cannot be reported for all ketones, even if it was reported in the case of thujone [86,87], as not all the alcohols have a sedative action, even if it was described for l-linalool [86,88,89]. 

**Figure 2:**
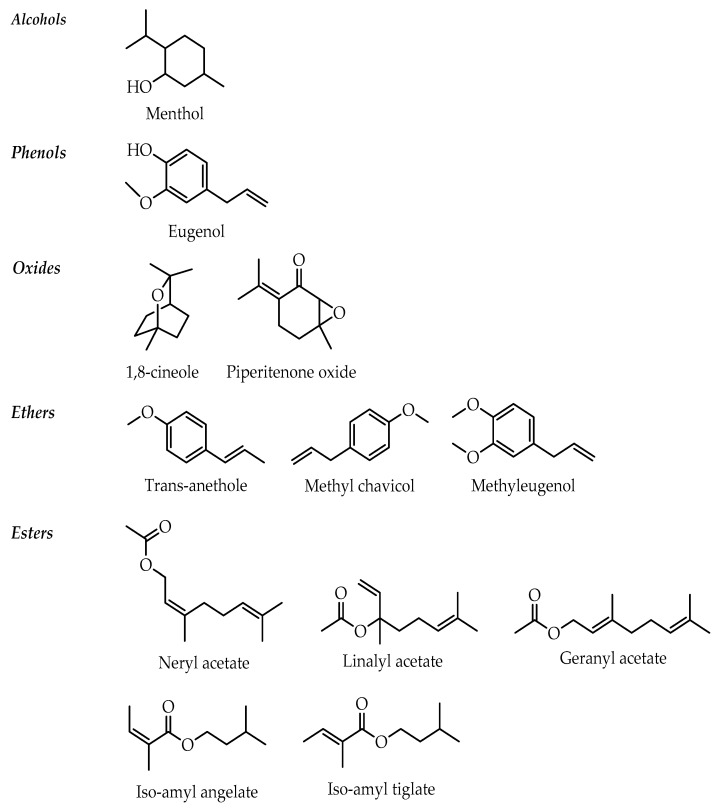
Chemical structures of main constituents from antispasmodic essential oils.

Several molecules with a structure clearly linked to the antispasmodic effect have been identified by our review. According to the surveyed literature, they are classified as alcohols (menthol), phenols (eugenol) [84], esters (linalyl acetate, neryl acetate, geranyl acetate, iso-amyl angelate and tiglate), ethers (trans-anethole, methyl chavicol or estragole, methyleugenol) [90], oxides (1,8-cineole, piperitenone oxide) [86,91,92] (Figure 2). However, other constituents present in low concentrations could be important for the pharmacological activity.

Chemical composition of the antispasmodic essential oils included in our study is presented in Table 3.

## 4. Conclusions

This review identified 39 plant species bearing essential oils with antispasmodic effect demonstrated in preclinical studies. The main mechanisms of the antispasmodic effect were represented by inhibition of voltage-dependent calcium channels, modulation of potassium channels and modulation of intracellular cAMP. Certain individual components identified in the chemical composition of the essential oils studied could become promising new drug candidates but future clinical studies are needed in order to ascertain their therapeutical value.

## Figures and Tables

**Table 1:** Plant species containing essential oils with antispasmodic activity demonstrated in preclinical studies.

No.	Plant Species with Essential Oils	Experimental Model/Concentration of EO in Organ Bath	Mechanism of Antispasmodic Effect	Reference
	Anacardiaceae			
1.	*Pistacia integerrima*—zebrawood	Isolated guinea pig ileum/50 μg/mL	Inhibition of Ca^2+^ channels	Shirole et al., 2015 [9]
	Annonaceae			
2.	*Cananga odorata var. genuina*—ylang ylang	Isolated rat bladder/0.05 mL/20 mL; white rabbit bladder in vivo/0.01–0.05 mL/rabbit ^∗^	Increase of cAMP	Kim et al., 2003 [10]
3.	*Xylopia frutescens*	Isolated guinea pig ileum/3–729 μg/mL	Inhibition of Ca^2+^channels; antagonism of histaminergic receptors	Souza et al., 2015 [11]
4.	*Xylopia langsdorffiana*	Isolated guinea pig ileum; isolated rat uterus/243–729 μg/mL	Decrease in cytosolic calcium concentration	Correia et al., 2015 [12]
	Apiaceae			
5.	*Anethum graveolens*—dill	Isolated rat ileum/0.5–2 mg/mL	Inhibition of Ca^2+^ channels	Gharib Naseri et al., 2007 [13]
6.	*Carum carvi*—caraway	Isolated guinea pig ileum/2.20–6.63 mg/mL; Dispersed smooth muscle cells of guinea pigs/2.5 mg/mL	Not available	Heinle et al., 2006 [14]; Al-Essa et al., 2010 [15]
7.	*Coriandrum sativum*—coriander	Isolated rabbit jejunum/ 1–30 mg/mL	Inhibition of Ca^2+^ channels	Jabeen at al., 2009 [16]
8.	*Ferula heuffelii* Griseb.	Isolated rat ileum/ 75–250 μg/mL	Not available	Pavlovic et al., 2012 [17]
9.	*Foeniculum vulgare*—fennel	Isolated rat uterus/10–40 mg/mL	Not available	Ostad et al., 2001 [18]
10.	*Pimpinella anisum*—aniseed	Isolated rat anococcygeus muscle/5–50 μg/mL	Activation of NO-cGMP pathway	Tirapelli et al., 2007 [19]
	Araceae			
11.	*Acorus calamus*—sweet flag, calamus	Isolated rabbit jejunum/ 0.3–1 mg/mL	Inhibition of Ca^2+^ channels	Gilani et al., 2006 [20]
	Asteraceae			
12.	*Artemisia dracunculus*—tarragon	Isolated sheep ruminal and abomasal smooth muscles/0.1–100 μg/mL	Not available	Jalilzadeh-Amin et al., 2012 [21]
13.	*Chamaemelum nobile*—roman chamomile	Isolated guinea pig ileum/60 μg/mL	Direct smooth muscle relaxation	Sandor et al., 2018 [22]
14.	*Chrysactinia mexicana*—damianita daisy	Isolated rabbit ileum/30 μg/mL	Inhibition of Ca^2+^ channels; increase of cAMP	Zavala-Mendoza et al., 2016 [23]
15.	*Hofmeisteria schaffneri*	Gastrointestinal transit test in mouse (in vivo)/316 mg/kg ^∗^	Not available	Perez-Vasquez et al., 2017 [24]
16.	*Matricaria recutita (chamomila)*—German chamomile	Isolated rabbit jejunum/0.3–3 mg/mL	K^+^ channels activation	Mehmood et al., 2015 [25]
	Geraniaceae			
17.	*Pelargonium graveolens*—geranium	Isolated guinea pig ileum/ 4.8–6 μg/mL	Reduction of calcium flux into the intestinal smooth muscles	Lis-Balchin et al., 1997 [26]
	Lamiaceae			
18.	*Lavandula angustifolia*—true lavender	Isolated guinea pig ileum, isolated rat uterus/6 μg/mL	Increase of cAMP	Lis-Balchin and Hart, 1999 [27]
19.	*Melissa officinalis*—melissa	Isolated rat ileum/20 μg/mL; isolated mouse jejunum/1–50 mg/mL	Inhibition of Ca^2+^ channels; Not available	Sadraei et al., 2003 [28]; Aubert et al., 2016 [29]
20.	*Mentha x piperita*—peppermint	Isolated guinea pig ileum; isolated rat ileum/10–320 μL/mL	Inhibition of Ca^2+^ channels; Inhibition of 5HT3 receptor channels	Grigoleit et al., 2005 [30]; Heimes et al., 2011 [31]
21.	*Mentha spicata*—spearmint	Isolated guinea pig ileum/0.1 nM–10 μM	Inhibition of Ca^2+^channels	Souza et al., 2013 [32]
22.	*Mentha x villosa*—mojito mint	Isolated guinea pig ileum/0.9 μM–2.5 μM	Not available	De Sousa et al., 2008 [33]
23.	*Ocimum basilicum*—basil	Isolated guinea pig ileum/3–10 mg/mL	Inhibition of Ca^2+^ channels	Janbaz et al., 2014 [34]
24.	*Ocimum selloi*—green pepperbasil	Isolated guinea pig ileum/250 μg/mL–1 mg/mL	Inhibition of Ca^2+^ channels	Souza et al., 2015 [35]
25.	*Ocimum gratissimum*—African basil	Isolated guinea pig ileum/0.1–1000 μg/mL	Not available	Madeira et al., 2002 [36]
26.	*Origanum majorana*—sweet marjoram	Isolated rabbit jejunum, isolated rat jejunum/0.01–0.3 mg/mL	Inhibition of Ca^2+^ channels	Makrane et al., 2018 [37]
27.	*Plectranthus barbatus* synonym *Coleus forskohlii*—Indian coleus	Isolated guinea pig ileum/1–300 μg/mL	Direct smooth muscle relaxation	Camara et al., 2003 [38]
28.	*Rosmarinus officinalis* —rosemary	Isolated guinea pig ileum/150–1200 μg/mL	Inhibition of Ca^2+^ channels	Ventura-Martinez et al., 2011 [39]
29.	*Salvia officinalis*—sage	Isolated rabbit jejunum/0.1–3 mg/mL	K^+^ channels activation	Khan et al., 2011 [40]
30.	*Satureja hortensis*—summer savory	Isolated rat ileum/1.55 μg/mL	Not available	Hajhashemi et al., 2000 [41]
	Poaceae			
31.	*Cymbopogon citratus*—lemongrass	Isolated rabbit ileum/0.001–1 mg/mL	Inhibition of Ca^2+^ channels	Devi et al., 2011 [42]
32.	*Cymbopogon schoenantus* (L.) Spreng.—camelgrass	Isolated rat ileum/30–120 μg/mL	Not available	Pavlovic et al., 2017 [43]
33.	*Cymbopogon martinii* —palmarosa	Isolated rabbit jejunum/0.01–3 mg/mL	Inhibition of Ca^2+^ channels	Janbaz et al., 2014 [44]
	Rosaceae			
34.	*Rosa indica* (L.)	Isolated rabbit jejunum/0.01–1 mg/mL	Inhibition of Ca^2+^ channels	Rasheed et al., 2015 [45]
	Rutaceae			
35.	*Citrus aurantifolia var. acida*—lime	Isolated rabbit jejunum/Not available	Not available	Spadaro et al., 2012 [46]
36.	*Citrus aurantium var. sinensis*—sweet orange	Isolated rat ileum/9.7–1000 μg/mL	Not available	Sanchez-Recillas et al., 2017 [47]
	Verbenaceae			
37.	*Lippia alba*	Isolated rat ileum/7–37 mg/mL	Reduction of calcium influx, stimulation of NO production	Blanco et al., 2013 [48]
38.	*Lippia thymoides*	Isolated guinea pig ileum/11.56–48.83 μg/mL	Not available	Menezes et al., 2018 [49]
	Zingiberaceae			
39.	*Elettaria cardamomum*—cardamom	Isolated rabbit jejunum/3–10 mg/mL	Inhibition of Ca^2+^ channels	Gilani et al., 2008 [50]

∗ For the in vivo experimental models dose of essentials oils (EO) was expressed in mL/animal or mg/kg.

**Table 2:** Clinical studies evaluating antispasmodic potential of essential oils.

Area of Interest	Authors	Type of Clinical Study	Number of Patients	Treatment	Results
Functional dyspepsia	Papathanasopoulos et al., 2013 [56]	Randomized, crossover study	13 healthy volunteers	Peppermint oil 182 mg p.o., single dose	Decreased intragastric pressure and gastric motility
Functional dyspepsia	Inamori et al., 2007 [57]	Randomized control study	10 healthy volunteers	Peppermint oil 0.64 mL p.o., single dose	Enhancement of gastric emptying without altering gastric emptying coefficient
Functional dyspepsia	May et al., 2000 [58]	Randomized control study	96 patients with functional dyspepsia	Peppermint oil and caraway oil combination 90 mg + 50 mg p.o., 4 weeks	Reduction of symptoms (pain, fulness, heaviness)
Functional dyspepsia	Madisch et al., 1999 [59]	Randomized control study	118 patients with functional dyspepsia	Peppermint oil and caraway oil combination 90 mg + 50 mg p.o., 4 weeks	Reduction of dyspeptic symptoms
Irritable bowel syndrome (IBS)	Cash et al., 2016 [60]	Randomized control study	72 patients with IBS	Peppermint oil 180 mg p.o., 4 weeks	Reduction of symptoms
IBS	Khanna et al., 2014 [61]	Meta-analysis	9 studies with 726 patients with IBS	Peppermint oil 200 mg	Global improvement of IBS symptoms (RR 2.23, 95% CI 1.78–2.81)
IBS	Merat et al., 2010 [62]	Randomized control study	90 patients with IBS	Peppermint oil 187 mg p.o., 8 weeks	Reduction of abdominal pain and discomfort
IBS	Cappello et al., 2007 [63]	Randomized control study	57 patients with IBS	Peppermint oil 225 mg p.o., 4 weeks	Reduction of total IBS symptoms
IBS	Pittler and Ernst 1998 [64]	Meta-analysis	8 randomized control studies	Peppermint oil	Reduction of IBS symptoms not established beyond reasonable doubt
IBS	Liu et al., 1997 [65]	Randomized control study	110 patients with IBS	Peppermint oil 187 mg p.o., 4 weeks	Improvement of pain and other IBS symptoms
Endoscopic procedures	Inoue et al., 2014 [66]	Randomized control study	226 patients with colonoscopy	L-menthol applied on the mucosa	Reduction of discomfort
Endoscopic procedures	Hiki et al., 2012 [67]	Randomized control study	131 patients with gastric endoscopy	L-menthol applied on the mucosa	Reduction of peristalsis
Endoscopic procedures	Yamamoto et al., 2006 [68]	Randomized, control study	40 patients with endoscopic cholangiopancreatography	Peppermint oil applied to papilla	Non-significant reduction of duodenal contractions
Infantile colic	Bezerra Alves et al., 2012 [69]	Randomized crossover study	30 infants	*Mentha piperita* liquid drops, 1 drop/kg	Decreased frequency and duration of infantile colic
Primary dysmenorrhea	Ghodsi and Asltoghiri, 2014 [70]	Randomized control study	80 female students	Fennel capsules 180 mg/day, 3 months	Reduction of dysmenorrhea symptoms

**Table 3:** Chemical composition of the studied antispasmodic essential oils.

Plant Species	Part Use	Representative Compounds	Reference
*Pistacia integerrima*, (Anacardiaceae)-*zebrawood*	Galls	Hydrocarbons: *monoterpenes*: α-pinene 21.81%, β-pinene 16.18, α-terpinene 1.37%, carene 11.09%, limonene 6.35%, α-phellandrene 15.48%, β-phellandrene 5.72%, *cis*-ß-ocimene 4.13%, *trans*-ß-ocimene 4.25%; *sesquiterpenes*: β-caryophyllene 3.88–5.33%, β-farnesene 7.88%; *aromatic*: *p*-cymene 11.54% Alcohols: terpinen-4-ol 11.93–28.82%, 4-carvomenthenol 17.06%, *p*-meth-1-en-8-ol 43.38%, borneol 8.90%, spathulenol 6.35% Ketones: tetrahydrocarvone 10.27% Esters: bornyl acetate 13.99%	[93,94,95]
*Cananga odorata var. genuina*, (Annonaceae)—ylang ylang	Flowers	Major components differ significantly depending on the fraction of essential oil, origin of the plant material and harvesting time Hydrocarbons: *sesquiterpenes*: ß-caryophyllene 15–26.8%, germacrene D 8.1–25.13%, δ-cadinene 2–4.7%, α-humulene 0.9–7.1%, α-farnesene 0.3–23.75% Alcohols: linalool 8.7–30%, farnesol 5.6% Ethers: p-methyl anisole 0.39–16.5% Esters: geranyl acetate 5–10%, farnesyl acetate 1–7%, methyl salicylate 1–10%, benzyl benzoate 3.8–27.48%, benzyl acetate 3–8%, methyl benzoate 1–6.05%	[86,96,97,98,99]
*Xylopia frutescens*, (Annonaceae)	Leaves	Hydrocarbons:*monoterpenes*: α-pinene 2.30%,β-ocimene 8.19%; *sesquiterpenes*: caryophyllene 23.91%, γ-cadinene 12.48%, γ-elemene 4.55%, β-elemene 4.31%, α-selinene 4.29%, δ-cadinene 3.02%, α-humulene 2.48%, γ-muurolene 2.23%, β-selinene 2.11% Alcohols: cadin-4-en-10-ol 5.78%, viridiflorol 4.83%, sphatulenol 3.97%	[11]
*Xylopia langsdorfiana*, (Annonaceae)	Fruits	Hydrocarbons:*monoterpenes*: α-pinene 37.73%, camphene 11.50%, β-pinene 4.04%, limonene 31.75%; *sesquiterpenes*: sclarene 10.38% Alcohols: α-terpineol 1.08%, spathulenol 1.74% Oxides: 1,8-cineol 1.15%, caryophyllene oxide 3.79%	[100]
*Anethum graveolens*, (Apiaceae)—dill	Seeds	Hydrocarbons: *monoterpenes*: limonene 1.11–83%, α-phellandrene *trace*–25%, β-phellandrene 0–3.38% Phenols: carveol 2%, eugenol Ketones: carvone (28–62.48%), *cis*-dihydrocarvone 0–5.87%, *trans*-dihydrocarvone 0–11.7%, piperitone 0–8.2% Ethers: apiole 0–16.79%, dillapiole 0–26.8	[86,101,102,103,104,105,106,107,108]
*Carum carvi*, (Apiaceae)—caraway	Fruits	Hydrocarbons: *monoterpenes*: limonene 1.5–51.3%, carvene 30% Alcohols: *cis*-carveol 5.5% Ketones: carvone 44.5–95.9% Ethers: *trans*-anethole 0–2.2%, apiole 12.3%	[86,109,110,111]
*Coriandrum sativum*, (Apiaceae)—coriander	Fruits	Hydrocarbons: *monoterpenes*: γ-terpinene 1–8%, limonene 0.1–4%, α-pinene 0–10.9%, ß-myrcene 0.2–2%; *aromatic p*-cymene *trace*–8.1% Alcohols: linalool 60–87%, geraniol 1.2–3.6%, terpinen-4-ol *trace*–3% Ketones: camphor 0.9–5.3% Esters: geranyl acetate 0.1–5.4%,linalyl acetate 0–2.7%	[86,89,112,113]
*Ferula heuffelii* Griseb., (Apiaceae)		Hydrocarbons: *monoterpenes*: α-pinene 4%, γ-terpinene 1.2%; *sesquiterpenes*: α-cadinene 3.4%, aromadendrene 1.8%, viridiflorene 2.1%, α-muurolene 1.7% Alcohols: viridoflorol 1.0%, cedrol 5.1% Ethers: myristicin 20.6%, elemicin 35.4% Esters: bornyl acetate 1.9%	[17]
*Foeniculum vulgare var. dulce*, (Apiaceae)—sweet fennel	Fruits	Hydrocarbons: *monoterpenes* α-pinene 0.4–10%, limonene 1.4–26.44%, α-phellandrene 0.2–9.26%, ß-myrcene 0.5–3%, ß-phellandrene 0.4–2.6%, γ-terpinene 10.5%, *cis*-ß ocimene 1.6–12%, α-terpinolene *trace*–3.3%; *aromatic*: *p*-cymene 0.1–4.7% Alcohols: fenchol *trace*–4% Ketones: fenchone *trace*–22% Ethers: methyl chavicol *trace*–17%, *cis*-anethole *trace*–1.7%, *trans*-anethole 50–90% Oxides: 1,8-cineole 1–6%	[86,114,115,116,117,118]
*Pimpinella anisum*, (Apiaceae) - aniseed	Fruits	Hydrocarbons: *sesquiterpenes*: γ-himachalene 0.4–8.2% Alcohols: anisol 0.5–4% Ethers: *cis*-anethole 0–1%, *trans*-anethole 90–93.7%, methyl chavicol 0–2.3% Aldehydes: anisaldehyde 0–5.4%	[86,119,120,121]
*Acorus calamus*, (Araceae)—*sweet flag, calamus*	Rhizomes	Hydrocarbons: *monoterpenes*: α-pinene 2.96%, limonene 0.1–2.8%; *sesquiterpenes*: ß-gurjunene 0.2–28.0%, calamenene 0.1–9.75%, δ-cadinene 0.5–2.1%, α-cedrene 3.09% Alcohols: linalool 0.3–12% Phenols: *cis*-isoeugenol 2.5–25%, *trans*-isoeugenol 0.5–2% Aldehydes: asaronal 0.2–6%, citronellal 2.82%, neral 2.57% Ketones: shyobunone *trace*–13.3%, epishyobunone 0.1–4.8%, isoshyobunone 0.6–13.0%, camphor 2.42% Ethers: methyl eugenol *trace*–8.59%, *cis*-methyl isoeugenol 2.4–49%, *trans*-methyl isoeugenol 1.1–7.9%, α-asarone 1–50.09%, ß-asarone 2.22–83.2%	[86,122,123,124,125,126]
*Artemisia dracunculus*, (Asteraceae)—tarragon	Flowering tops and leaves	Major components differ significantly depending on the origin of the plant material and harvesting time Hydrocarbons: *monoterpenes*: α-pinene 5.1%, limonene 2.40–12.4%, *trans*-ocimene 2.99–20.6%, α-terpinolene 0.5–25.4%, cis-ocimene 2.65 –22.2% sabinene 14.28–39.44% Ethers: *trans*-anethole 10–21.2%, *cis*-anethole 53.37–81.0%, methyl eugenol 2.2–39.35%, methyl isoeugenol 1.8–35.8%, methyl chavicol 1.09–74.46% Others: asarone 21.69–40.36	[127,128,129,130,131,132]
*Chamaemelum nobile* (Asteraceae)—roman chamomile	Flowers	Hydrocarbons: *monoterpenes*: α-terpinene 0–10%, α-pinene 0–10%, ß-pinene 0–10%, sabinene 0–10%; *sesquiterpenes*: caryophyllene 0–10% Alcohols: *trans*-pinocarveol 5% Aldehydes: myrtenal 0–10% Ketones: pinocarvone 13% Oxides: 1,8-cineole 0–25% Ethers: methyl chavicol 5% Esters: 2-methylbutyl 2-methyl propionate 0.5–25%, 2-methylpropyl butanoate 0.5–10%, 2-methylbutyl, 2-methylbutanoate 0.5–25%, 2-methylpropyl 3-methylbutanoate 0–10%, propyl angelate 0.5–10%, 2-methylpropyl angelate 0.5–25%, butyl angelate 0.5–10%, 3-methylpentyl angelate 0–10%, isobutyl angelate 36–40%, isobutyl isobutanoate 4%, 2-methylbutyl methyl-2-butanoate 3%, isoamyl methyl-2 butanoate 3%, hexyl acetate 0.5–10%	[86,133,134,135,136,137,138]
*Chrysactinia mexicana*, (Asteraceae)—damianita daisy	Leaves	Hydrocarbons: *monoterpenes*: α-myrcene 1.20% Alcohols: linalool 1.39% Ketones: α-thujone 1.17%, piperitone 37.74% Oxides: 1,8-cineole 41.3% Esters: linalyl acetate 9.08%	[139]
*Hofmeisteria schaffneri* (Asteraceae)	Aerial parts	Major components differ depending on the harvesting time Alcohols: linalool 0.25–1.38% Esters: thymyl isobutyrate 1.54–3-41%, thymyl isovalerate 14.12–30.97%, 9-acetoxy-8,9-dehydrothymyl angelate 2.36–5.23%, 8,9-epoxy-10-acetoxythymyl angelate 0.41–15% Others: hofmeisterin III 24.12–34.85%	[140]
*Matricaria recutita* (Asteraceae)—german chamomile	Flowers	Hydrocarbons: *sesquiterpenes* chamazulene 1–35%, *trans*-ß-farnesene 2–13%, *trans*-α-farnesene 27%, δ-cadinene 5.2%, γ-muurolene 1.3%, α-muurolene 3.4% Alcohols: *sesquiterpenols*: α-bisabolol 2–67% Oxides:α-bisabol oxide A 0–55%, α-bisabolol oxide B 4.3–19%, bisabolone oxide A 0–64%	[127,133,134,135]
*Pelargonium graveolens*, (Geraniaceae)—geranium	Aerial parts	Hydrocarbons: *monoterpenes*:α-pinene 22.47%; *sesquiterpenes*: guai-6,9-diene 3.9–5.3%, β-bourbonene 2.7–3.14%, germacrene D 2.92–4.33%, γ-cadinene 2.38% Alcohols: citronellol 15.2–48.44%, geraniol 6–25%, linalool 1–13.79%, octen-1-ol 18.61% Aldehydes:geranial 0–9% Ketones: menthone 0.6–6.96%, isomenthone 4–8.4% Oxides: *cis*-rose oxide 0.69–25%, *trans*-rose oxide 0.31–2.01%, cariopyllene oxide 2.52–3.7% Esters: citronellyl formate 8–24.4%, geranyl formate 1–6.22%, citronellyl propionate 1–3%, geranyl angelate 1–2%, citronellyl butanoate 1.3%, geranyl butanoate 1.3%	[86,99,141,142,143,144]
*Lavandula angustifolia* (Lamiaceae)—true lavender	Flowers	Hydrocarbons: *monoterpenes*:*cis*-ß-ocimene 1.3–10.9%, *trans*-ß-ocimene 0.8–5.8%, limonene 0.2–7%; *sesquiterpenes*: ß-caryophyllene 2.6–7.6% Alcohols: linalool 26–49%, terpinen-4-ol 0.03–6.4%, α-terpineol 0.1–1.4%, borneol 0.8–1.4%, lavandulol 0.5–1.5% Oxides: 1,8-cineole 0.5–2.5% Esters: linalyl acetate 35–55%, lavandulyl acetate 0.2–5.9%	[33,89,99,137,145,146,147,148]
*Melissa officinalis* (Lamiaceae)—melissa	Aerial parts	Major components differ significantly depending on the origin of the plant material Hydrocarbons: *sesquiterpenes*: ß-caryophyllene 8–10%, α-copaene 4–5% Alcohols: linalool 0.4–2.74%, nerol 1.4%, geraniol 0.20-27.22%, citronelol 0-36.71% Aldehydes: neral 3.28–31.5%, geranial 0-38.13%, citronellal 1.48–39.6% Oxides: caryophyllene oxide 0.2–10.26%	[149,150,151,152]
*Mentha × piperita* (Lamiaceae)—peppermint	Aerial parts	Hydrocarbons: *monoterpenes*: α-pinene 0.2–2%, ß-pinene 0.3–4%, limonene 0.6–6%; *sesquiterpenes*: germacrene D 1.75–4.3% Alcohols: menthol 25.16–48%, neomenthol 2–7.7%, α-terpineol 0.1–1.9%, *cis*-carveol 3.35%, terpinen-4-ol 0–2.4%, *cis*-thujan-4-ol 0.2–1.4%, viridiflorol 0.5–1.3%, Ketones: menthone 16–42.97%, isomenthone 4–10.4%, neomenthone 2–3%, piperitone 0.5–1.2%, pulegone 4.39% Oxides: 1,8-cineole 2.15–7.4%, transpiperitonoxide 0.5–3.1% Esters: menthyl acetate 1.6–10% Benzofurans: menthofuran 0.1–5.7%	[153,154,155,156]
*Mentha spicata* (Lamiaceae)—spearmint	Aerial parts	Hydrocarbons: *monoterpenes*: ß-pinene 0.3–2.3%, ß-myrcene 1.2–5.5, limonene 2–25%; *sesquiterpenes*: ß-caryophyllene 0.3–4.41%, β –farnesene 1.71%, ß-bourbonene *trace*–2.14%, germacrene D 0–3.14% Alcohols: *cis*-carveol 5.30%, menthol 0.5–2%, terpinen-4-ol *trace*–6.1%, α-terpineol 0–2.7% Ketones: carvone 39–70%, menthone *trace*–5.2%, *cis*-dihydrocarvone 3.1–21.6%, *trans*-dihydrocarvone 0–21%, isomenthone 3.33% Oxides: 1,8-cineole 0.5–17.0%, piperetenone oxide *trace*—79.2% Esters: dihydrocarvyl acetate 1.2–24.8%, *cis*-carvyl acetate 0.2–5.5%, *trans*-carvyl acetate 0.7–5.9%, neoisodihydrocarveol acetate 0–21%, menthyl acetate 2% Benzofurans: menthofuran 2%	[86,99,156,157,158]
*Mentha x vilosa* Huds.(Lamiaceae)—mojito mint	Aerial parts	Hydrocarbons: *monoterpenes*:ß-pinene 1.42–4.04%, myrcene 3.10–3.66%, limonene 2.38–8.75%; *sesquiterpenes*: ß-caryophyllene 2.82–5.16%,δ-cardinene 9.69%, γ-muurolene 2.18–16.02%, germacrene-D 3.81% Oxides: 1,8-cineole 1.58–3.93%, piperitenone oxide 58.74–79.03%, cariophyllene oxide 2.82%	[33,91,159,160,161]
*Ocimum basilicum* (Lamiaceae)—basil	Aerial parts	Hydrocarbons: *sesquiterpenes*:ß-caryophyllene 2–3% Alcohols: linalool 40–55%, α-fenchyl alcohol 3–12%, terpinen-4-ol 1.6%, α-terpineol 2% Phenols: eugenol 1–19%, iso-eugenol 2% Oxides: 1,8-cineole 2–8% Ethers: methyl chavicol 3–31%, methyl eugenol 1–9% Esters: methyl cinnamate 0.1–7%	[86,162]
*Ocimum selloi* (Lamiaceae) —green pepper basil	Aerial parts	Hydrocarbons: *sesquiterpenes*:ß-caryophyllene 2.2–3%, germacrene D 0–3.14% Alcohols: linalool 20.6%, spathulenol 1.3% Ethers: *trans*-anethole 45.42%, *cis*-anethole 3.95%, methyl chavicol 24.14–93.2%, methyl eugenol 2.2-39.35%	[35,162,163,164]
*Ocimum gratissimum* (Lamiaceae)—african basil	Aerial parts	Hydrocarbons: *monoterpenes*:β-pinene 6.2%, *cis*-ocimene 13.9–23.97%, *trans*-ocimene 19.60–48.28%, γ-terpinene 0.20–28.10%, limonene 11.40%; *sesquiterpenes*: ß-caryophyllene 2.7–3.06%, β-phellandrene (21.10), germacrene D 7.30-10.36%, α-*trans*-bergamotene 4.1%, γ- muurolene 9.32–11.6%; *aromatic*: *p*-cymene 4.40–19.90% Phenols: eugenol 10.70–74.80%, thymol 13.10–46.60% Oxides: 1,8-cineole 0–54.94%	[36,162,165,166]
*Origanum majorana*, (Lamiaceae)—sweet marjoram	Aerial parts	Major components differ significantly depending on chemotype and the origin of the plant material Hydrocarbons: *monoterpenes*: sabinene 1.45–10%, ß-myrcene 1–9%, α-terpinolene 1–7%, α-pinene 1–5%, *cis*-/*trans*-ß-ocimenes 6.4%, 3-carene 6.2%, myrcene 1.12-4.7%, α-terpinene 3.9–8%, γ-terpinene 11.16–20%; *sesquiterpenes*: ß-caryophyllene 2–7.44%, δ-cadinene 4.2%, α-farnesene 4.58%, germacrene D 9.2%; *aromatic*: benzene 13.34%, *p*-cymene 7.0–12.05% Alcohols: terpinen-4-ol 14–38.4%, *cis*-thujan-4-ol 0.11–44%, *trans*-thujan-4-ol 1–5%, linalool 2–31.68%, α-terpineol 7–27% Phenols: carvacrol 0–83.47% Esters: terpenyl acetate 0–3%, geranyl acetate 1–7.8%, linalyl acetate 2.41–17.4%	[86,167,168,169,170,171]
*Plectranthus barbatus* synonym *Coleus forskohlii*, (Lamiaceae)—*Iindian coleus*	Aerial parts	Hydrocarbons: *monoterpenes*: α-pinene 12–67%,β-pinene 0.1-22%, β-myrcene 1.8%, *cis*-β-ocimene 1.9%, *trans*-β-ocimene 1.2%; *sesquiterpenes*: β-caryophyllene 7-12%, α-copaene 8.9%, β-cubebene 3.7% Alcohols: oct-1-en-3-ol *traces*–28% Phenols: thymol 15.3%, carvacrol 12.1%, eugenol 25.1%	[38,172,173,174]
*Rosmarinus officinalis* ct. verbenone, (Lamiaceae)—rosemary	Aerial parts	Hydrocarbons: *monoterpenes*: α-pinene 15–34% Alcohols: borneol *trace*–16.63% Ketones: verbenone 15–37%, camphor 1–22.35% Oxides: 1,8-cineole *trace*–20% Esters: bornyl acetate 12%	[86,175,176,177]
*Salvia officinalis* (Lamiaceae)—*sage*	Aerial parts	Hydrocarbons: *monoterpenes*: α-pinene 2.02–6.4%, ß-pinene 1.9–8.20%, camphene 1–8.49%, ß-myrcene 0.4–5.66%, limonene 0.9–4%; *sesquiterpenes*: ß-caryophyllene 1–7%, α-humulene 1.6–5% Alcohols: linalool 0.4–12%, terpinen-4-ol 0.2–4%, α-terpineol *trace*–9%, borneol 1.5–14%, viridiflorol 0–10% Ketones:α-thujone1.1–35.7%,ß-thujone 1.71–33%, camphor 4.1–43.83% Oxides: 1,8-cineole 5–57.18%, caryophyllene oxide 0.4–2.1% Esters: bornyl acetate 0.1–5.59%, linalyl acetate 1–2%	[40,86,178,179]
*Satureja hortensis* (Lamiaceae)—summer savory	Aerial parts	Hydrocarbons: *monoterpenes*: α-terpinene 1.29-3.1%, γ-terpinene 12.8-24%, β-myrcene 1–2.8%; *sesquiterpenes*: ß-caryophyllene 1.2–4%, δ-cadinene 3%; *aromatic*: *p*-cymene 3.7–20% Alcohols: linalool 9–54%, terpinen-4-ol *trace*–7%, α-terpineol 6–9% Phenols: carvacrol 59.70–67.00%, eugenol 1–1.7%, thymol 0-29.0% Oxides: 1,8-cineole 0-37.82%	[86,99,180,181]
*Cymbopogon citratus***Stapf**(Poaceae)—West Indian lemongrass	Aerial parts	Hydrocarbons: *monoterpenes*: limonene 2.4–2.6%, β-myrcene 2.34–21% Alcohols: α-terpineol 0.2–2.3%, linalool 1.2–3.4%, geraniol 2.6–40%, nerol 0.8–4.5%, citronellol 0.1–8%, farnesol 12.8% Aldehydes: neral 3–43%, geranial 4.5–58%, citronellal 0.1–9% Esters: geranyl acetate 0.1–3.0%	[42,86,182,183,184,185]
*Cymbopogon schoenantus (L.)* Spreng (Poaceae)—camelgrass	Aerial parts	Hydrocarbons: *monoterpenes*: limonene 1.5–3.12%; *sesquiterpenes*: β-elemene 11.6%, δ-2-carene 10% Alcohols: α-eudesmol 11.5%, elemol 10.8%, β-eudesmol 8.5%, γ- eudesmol 4.2%, intermedeol 6.1–17.3%, linalool 21.6% Aldehydes: neral 3.3%, geranial 2.4% Ketones: piperitone 47.7–71.5%	[43,185]
*Cymbopogon martini* (Poaceae)—palmarosa	Aerial parts	Alcohols: linalool 1.6–3.4%, geraniol 67.6–83.6%, citronellol 1.6–2.1% Aldehydes: geranial 1–8.8% Esters: geranyl acetate 2.2–24.6%	[185,186]
*Rosa indica L*, (Rosaceae)		*Hydrocarbons*: nonadecane 3.4%, (Z)-9-nonadecene 3.3%, heneicosane 6.7%, tricosane 5.6%, pentacosane 4.9% Alcohols: citronellol 11.7%, nerol 8.0%, geraniol 24.8%, farnesol 2.0%	[45,187]
*Citrus aurantifolia*/*Citrus medica var. acida* (Rutaceae)—lime	Pericarps	Hydrocarbons: *monoterpenes*: limonene 36–60%, γ-terpinene 6–17.6%, α-pinene 0.2–5.03%, ß-pinene 4.9–19.5%, ß-myrcene 1–2.6%; *sesquiterpenes*: ß-caryophyllene 1.3–3.4%, α-bisabolene 2.3%; *aromatic p*-cymene 0.1–6.8% Alcohols: linalool 1.4–16.9%, α-terpineol 13–23% Aldehydes: citronellal 0–5.3%, neral 0.7–4.7%, geranial 1.81–6.4% Esters: linalyl acetate 26–27%	[46,86,99,188,189]
*Citrus aurantium var. sinensis* (Rutaceae)—sweet orange	Pericarps	Hydrocarbons: limonene 87.9–96.8%, ß-myrcene 1.37–2.5%, ß-phellandrene 0–1.5% Alcohols: linalool 0.5–2.4% Ketones: carvone 1.8%	[86,99,190,191]
*Lippia alba*, (Verbenaceae)	Leaves	The plant presents a great morphological and chemical variability with a predominance of monoterpene type compounds such as citral, β-myrcene, limonene and carvone, based on which several chemotyps have been described. Hydrocarbons: *monoterpenes*: limonene 8.2–15.7%, γ-terpinene 4.09%, myrcene 6.6–8.3%; *sesquiterpenes*: β-caryophyllene 2.7–3.07%, germacrene D 3.0–5.47% Alcohols: β-elemol 5.37%, nerol 2.2%, geraniol 2.9%, linalool 0.8–64.2% Aldehydes: geranial 6.5–50.94%, neral 11.5–33.32% Ketones: carvone 16.7–33.7% Oxides: cariopyllene oxide 0–2.64%	[48,192,193,194,195]
*Lippia thymoides*, (Verbenaceae)	Leaves	Hydrocarbons: *monoterpenes*: α-pinene 0.94–2.38%, camphene 2.64–5.66%, limonene 1.67–3.75%,; *sesquiterpenes*: copaene 2.42–3.38%, β-caryophyllene 5.32–26.27%, α-caryophyllene 3.06-5.48%, germacrene D 4.72–6.18% Alcohols: borneol 4.45–7.36% Phenols: thymol *trace*–66.33% Ketones: camphor 3.22–8.61% Oxides: cariopyllene oxide 0.9–2.7% Ethers: 1,8-cineole 1.86–4.5% Esters: thymol acetate 0-7.49%	[49,196,197]
*Elettaria cardamomum*, (Zingiberaceae)—cardomom	Fruits	Hydrocarbons: *monoterpenes*: limonene 1.7–14%, sabinene 1.3–5%, ß-myrcene 0.2–2.2% Alcohols: linalool 0.4–6.9%, terpinen-4-ol 0.1–3.2%, α-terpineol 0.8–5.25%, geraniol 0.2–1.6%, *trans*-nerolidol 0.1–2.7%, *cis*-nerolidol 0.2–1.6% Oxides: 1,8-cineole 15.13–50% Esters: α-terpinyl acetate 29–56.87%, linalyl acetate 0.2–7.7%	[86,198,199,200,201,202]
